# Is there a ‘bipolar iceberg’ in UK primary care psychological therapy services?

**DOI:** 10.1017/S0033291722002343

**Published:** 2023-09

**Authors:** Rebecca Strawbridge, Laith Alexander, Thomas Richardson, Allan H. Young, Anthony J. Cleare

**Affiliations:** 1 Department of Psychological Medicine, Institute of Psychiatry, Psychology & Neuroscience, King's College London, London, UK; 2 South London & Maudsley NHS Foundation Trust, London, UK; 3 School of Psychology, University of Southampton, Southampton, UK

**Keywords:** Bipolar disorders, bipolar spectrum, major depression, primary care, psychological therapy

## Abstract

**Background:**

*Improving Access to Psychological Therapies* (IAPT) is a primary care therapy service commissioned by England's National Health Service (NHS) for people with unipolar depression and anxiety-related disorders. Its scope does not extend to ‘severe mental illness’, including bipolar disorders (BD), but evidence suggests there is a high BD prevalence in ostensibly unipolar major depressive disorder (uMDD) samples. This study aimed to indicate the prevalence and characteristics of people with BD in a naturalistic cohort of IAPT patients.

**Methods:**

371 participants were assessed before initiating therapy. Participants were categorised by indicated diagnoses: BD type-I (BD-I) or type-II (BD-II) as defined using a DSM diagnostic interview, bipolar spectrum (BSp, not meeting diagnostic criteria but exceeding BD screening thresholds), lifetime uMDD or other. Information about psychiatric history and co-morbidities was examined, along with symptoms before and after therapy.

**Results:**

368 patients provided sufficient data to enable classification. 10% of participants were grouped as having BD-I, 20% BD-II, 40% BSp, 25% uMDD and 5% other. BD and uMDD participants had similar demographic characteristics, but patients meeting criteria for BD-I/BD-II had more complex psychiatric presentations. All three ‘bipolar’ groups had particularly high rates of anxiety disorders. IAPT therapy receipt was comparable between groups, as was therapy response (*F*_9704_ = 1.113, *p* = 0.351).

**Conclusions:**

Notwithstanding the possibility that bipolar diathesis was overestimated, findings illustrate a high prevalence of BD in groups of people notionally with uMDD or anxiety. As well as improving the detection of BD, further substantive investigation is required to establish whether individuals affected by BD should be eligible for primary care psychological intervention.

## Introduction

Bipolar disorders (BD) and unipolar major depressive disorder (uMDD) are two of the most disabling health conditions globally as measured by the number of healthy life years lost (GBD, 2019 Mental Disorders Collaborators, 2022). The high morbidity and mortality can be reduced by intervening early in the course of illness (Vieta et al., 2018). However, access to appropriate treatment is a particular problem in people with BD. One reason for this is the extensive diagnostic delays with high rates of misdiagnosis leading to inappropriate pharmacological treatment (Hirschfeld, Lewis, & Vornik, 2003). A second reason is that psychological therapies are underutilised (Jones et al., 2018) despite being highly recommended in guidelines (NICE, 2020).

England's National Health Service (NHS) implemented the Improving Access to Psychological Therapies (IAPT) programme in 2008, as the world's first freely accessible national psychological therapy service. The IAPT service is locally funded by clinical commissioning groups. Generally, individuals can self-refer or be referred by a primary/secondary care physician; are triaged, screened and formally diagnosed to determine suitability for the service; and receive evidence-based psychological therapy at the appropriate dose. The IAPT programme is currently not available to people with ‘severe mental illness’ (SMI) including BD (Jones et al., 2018), although there is a lack of data actually examining rates of BD in people who have received IAPT treatment. The IAPT for Severe Mental Illness (IAPT-SMI) initiative has tried to increased access for bipolar disorder, with current pilot sights in South London and Maudsley NHS Trust demonstratingpromising findings about good engagement and clinical outcomes (Johns et al., 2019). A recent survey in the UK found that only 15% of those with the diagnosis of BD had ever been offered therapy by an IAPT service (Bipolar UK, 2022). Considering the reported high rates of undiagnosed BD in samples with common mental health conditions (Angst et al., 2011) and the notional unsuitability of IAPT for these individuals, this naturalistic study sought to identify the prevalence of bipolar spectrum disorders in a cohort of IAPT referees; their clinical characteristics; and their subsequent response to therapy.

## Objectives

Specifically, this study examined data from a naturalistic observational investigation of individuals referred to an IAPT service in South London, seeking to meet three aims:

1. *Measuring the prevalence of bipolar spectrum disorders in a cohort of IAPT referees*. Based on prior data, the rate of BD (type I/II combined) was hypothesised to be approximately 16% and bipolar spectrum to be an additional 31% (Angst et al., 2011).
2. *Characterising socio/demographic and clinical features of participants with bipolar* (*spectrum*) *disorders.* It was hypothesised that although participants with BD may differ by subtype, differences between these participants and those with uMDD would be more substantial (Angst et al., 2011).
3. *Therapeutic characteristics of participants with bipolar* (*spectrum*) *disorders.* No specific hypotheses were specified for this objective, in the absence of relevant previous evidence.

All of the above compare participants according to pre-defined groups (see Measures for definitions) i.e., BD type one (BD-I), BD type two (BD-II), non-DSM bipolar spectrum (BSp) and uMDD. Most participants were expected to have experienced a major depressive episode.

## Methods

### Design

The PRedicting OutcoMe following Psychological Therapy in IAPT (PROMPT) study was an observational longitudinal investigation of individuals referred to one South London IAPT service (Southwark). The primary study assessed (prior to initiating therapy) a range of factors putatively predicting subsequent response to naturalistic IAPT intervention. Full details of the study are described elsewhere (Grant et al., 2014; Hepgul et al., 2016; Strawbridge et al., 2020).

### Procedures

Approval was obtained from the Bromley NHS Research Ethics Committee (13/LO/1347) and participants were recruited between February 2014 until July 2016. Upon referral to the IAPT clinical service all patients are, as standard practice, asked for their consent to be contacted for research studies. This takes place before any clinical activities. Contact details for consenting individuals were provided to the PROMPT research team and these potential participants were then contacted by study researchers, who provided information and invited participation. The study was not advertised as relating to bipolar disorder. All eligible and willing individuals provided informed consent prior to participation. Study participation comprised a single research visit prior to starting therapy, where all non-therapy data were collected. Records collected as standard by the IAPT service were recorded on a longitudinal basis as participants continued their naturalistic treatment through the service. Therapy outcomes were taken from the last therapy session attended. This paper describes a secondary analysis of PROMPT data.

### Participants

The only eligibility criteria required participants to have the capacity and willingness to consent to participate; to be ⩾18 years old; and planning to engage in IAPT therapy. As a naturalistic observation of individuals in IAPT, participants were included regardless of current diagnosis or symptoms.

### Measures

#### Diagnostic subgroups

The Mini-International Neuropsychiatric Interview (MINI; Sheehan et al., 1998) is a structured diagnostic interview reflecting the current (at the time the study was undertaken) DSM-IV criteria, administered in this study by trained researchers. Participants meeting MINI criteria for BD-I or BD-II were categorised as such. Participants not meeting these criteria but who exceeded the standardised score threshold (⩾8) on the patient-rated 16-item Hypomanic CheckList (HCL) screening tool (indicating a high likelihood of having a BD) were categorised as having BSp. The HCL has been reported as having an excellent ability to distinguish between BD and MDD from its validation against clinician-confirmed diagnoses (Forty et al., 2010). Those not meeting any of the aforementioned BD criteria were otherwise categorised as uMDD (if meeting MINI criteria for a lifetime major depressive episode) or otherwise nMDD (e.g. anxiety disorders). The diagnostic-related characteristics of the latter group are narratively summarised. See Online Supplement 1 for full details of measures administered.

#### Sociodemographic & clinical characteristics

Measures were pre-selected for inclusion based on existing evidence of associations with bipolarity in addition to data availability from the PROMPT study. Sociodemographic variables comprised age, gender, ethnicity, education, BMI, physical illness severity, social support, relationship and employment statuses. Historical clinical characteristics included past episodes of MDD, recurrent MDD, psychosis, age of mental illness onset, hospitalisations, negative life events (lifetime and recent) and childhood trauma. Current presentation factors included comorbidities (obsessive compulsive disorder [OCD], post-traumatic stress disorder [PTSD], generalised anxiety disorder [GAD], other anxiety disorders, substance or alcohol abuse, eating disorders) as well as alcohol intake, traits of borderline personality disorder (BPD) and personality disorders more broadly, level of suicidality, self-criticism, illness perception and psychotropic medication use.

#### Therapy characteristics

As well as the proportion of participants receiving therapy (defined as attending ⩾2 sessions), the number of sessions and type of therapy received, responses to therapy were assessed using baseline and post-therapy scores on participant-rated symptoms of depression (PHQ-9), anxiety (GAD-7) and psychosocial functioning (WSAS).

### Data analysis

In addition to descriptive examination of results, the following analyses (all using in SPSS v26) were undertaken in accordance with the study's objectives:

Objective 1: To estimate the prevalence of bipolar (spectrum) disorders, raw percentages for each pre-determined group were calculated.

Objective 2: To estimate differences in the characteristics of each group, initial univariate tests were undertaken: Comparisons of continuous variables were examined using a four-way ANOVA with between-group comparisons (BD-I *v.* BD-II *v.* BSp *v.* uMDD) corrected using Tukey's honestly significant difference. For categorical variables, chi-square or Fisher's exact test was used, with between-group comparisons corrected using Bonferroni correction. Subsequently, multinomial logistic regression analysis compared BD-I and BD-II (as an aggregate) *v.* BSp *v.* uMDD as dependent variables. Independent variables were included in the model as per indications from univariate analyses, excluding those that were highly collinear (Pearson's *r* > 0.400) or had few subjects (<10) in any category. This approach attempted to maximise statistical power to detect differences while maintaining examination of the most relevant factors, to estimate the amount of variance between groups that could be explained by such characteristics.

Objective 3: To indicate therapeutic differences, the rate of therapy receipt was compared between groups using chi-square and Kruskall-Wallis tests. For therapy recipients, the type of therapy received was compared using the same approach. The response to therapy was examined using MANOVA comparing the three therapy outcome measures (PHQ-9, GAD-7, WSAS) before and after treatment, between the four participant groups.

## Results

Of the 371 patients assessed, 368 provided sufficient data to enable classification into BD-I, BD-II, BSp, uMDD or nMDD groups. Age was the only demographic characteristic to differ between groups (*F*_4,363_ = 3.226, *p* = 0.013) with nMDD patients significantly older than BD-II (*p* = 0.018) and BSp (*p* = 0.034); see Table 1. Mean (s.d.) HCL scores between groups were BD-I 8.82 (3.24), BD-II 10.41 (2.48), BSp 9.93 (1.53) and uMDD 4.92 (2.09). The proportions of patients currently meeting MINI criteria for a current major depressive episode were BD-I 63%, BD-II 53%, BSp 53% and uMDD 59%.

**Table 1.** Sociodemographic characteristics

Characteristic		*n*	ALL *N* = 368	BD-I *n* = 35	BD-II *n* = 75	BSp *n* = 147	uMDD *n* = 91	nMDD *n* = 20
Age	Mean (s.d.)	368	39.6 (13.1)	37.9 (12.0)	37.5 (12.9)	38.7 (13.6)	41.5 (11.8)	47.6 (14.7)
Gender	Female	368	230	21	38	96	61	14
	Male		138	14	37	51	30	6
Ethnicity	White	287	222	25	46	83	55	13
	Black		23	1	6	6	10	0
	Asian		6	1	3	1	1	0
	Mixed		14	2	2	8	2	0
	Other		22	4	3	9	5	1
BMI	Mean (s.d.)	347	25.3 (7.1)	23.4 (4.1)	24.6 (8.7)	25.6 (7.0)	25.7 (6.5)	26.1 (6.5)
Physical illness score	Mean (s.d.)	298	15.6 (2.7)	15.5 (3.9)	15.4 (2.4)	15.6 (2.8)	15.8 (2.7)	15.0 (2.0)
Education level (highest qualification)	None	342	31	5	2	12	11	1
	GCSE/O-level		60	6	13	18	20	3
	A-level/GNVQ		73	8	17	33	12	3
	Higher degree or above		178	14	39	76	39	10
Relationship status	Single	285	135	12	37	51	29	6
	Separated/divorced		23	0	3	12	7	1
	Steady/married		127	10	22	55	32	8
Employment status	Working/studying	341	214	18	43	94	50	9
	Not working		127	16	27	45	32	7
Social support score	Mean (s.d.)	343	9.9 (2.4)	9.5 (2.4)	9.8 (2.7)	10.0 (2.1)	9.7 (2.6)	10.2 (2.1)

BD-I, bipolar disorder type 1; BD-II, bipolar disorder type 2; BSp, bipolar spectrum; uMDD, unipolar major depressive disorder; nMDD, not meeting criteria for either bipolar disorders or unipolar MDD; s.d., standard deviation; BMI, body mass index; GCSE, General Certificate of Secondary Education; O-level, Ordinary-level; A-level, Advanced-level; GNVQ, General National Vocational Qualification.

Sociodemographic characteristics as per participant report, except physical illness severity (total Modified Cumulative Illness Rating Scale score excluding the mental health item) and social support extent (total Oslo 3 social support scale score).

### Prevalence

**Fig. 1.**
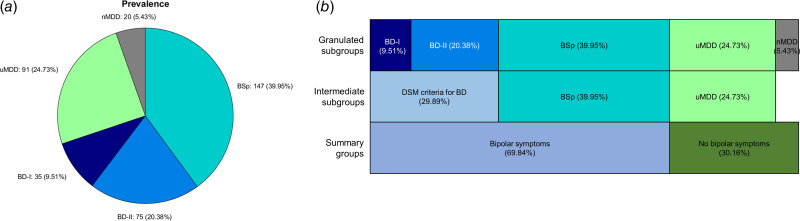
Prevalence of bipolar symptomatology in 368 people presenting to an Improving Access to Psychotherapy (IAPT) service. (**A):** Using the Mini-International Neuropsychiatric Interview (MINI), 9.5% of people met the criteria for Bipolar Disorder type one (BD-I) and 20.4% of people met the criteria for Bipolar Disorder type two (BD-II). The modal group – 39.9% of people – did not meet the MINI-defined criteria for BD, but exceeded the threshold on the 16-item Hypomanic CheckList (HCL), termed bipolar spectrum (BSp). 24.7% of people met the criteria for unipolar Major Depressive Disorder (uMDD), and 5.4% of people did not meet the criteria for BD or uMDD (termed no MDD, or nMDD). (**B):** The top row shows the percentages of people in each group as depicted in A (granulated subgroups). In total, 29.9% of people met DSM criteria for BD (intermediate subgroups), and 69.8% of patients had some degree of bipolar symptomatology (summary groups).

As displayed in Fig. 1, 9.5% of patients (*n* = 35) met MINI-defined criteria for BD-I and 20.4% (*n* = 75) met MINI-defined criteria for BD-II. An additional 39.9% (*n* = 147) were classified as BSp. Overall, 69.8% met our criteria for a bipolar (spectrum) disorder.

24.7% (*n* = 91) met the criteria for uMDD, and 5.4% (*n* = 20) were not eligible for classification into the aforementioned groups (nMDD). The nMDD group (not included in subsequent analyses) were heterogeneous, with most individuals meeting the criteria for a current anxiety disorder (plus 1 with alcohol use disorder, 4 with previous panic disorder and 4 not meeting any of the assessed diagnostic criteria).

### Characteristics

#### Univariate analyses

Patients meeting either BD-I or BD-II criteria had more prolonged and extensive past psychiatric histories (Table 2). In addition to indications of significant overall effects, specific group differences that maintained significance (*p* < 0.05) after multiple comparisons corrections are noted below, in Table 2 and Online Supplement 2.

**Table 2.** Clinical characteristics

Characteristic		*n*	ALL	BD-I	BD-II	BSp	uMDD	Overall effect, *p* value	Significant between-group comparisons
Past depression (MINI)	*n* (%) yes	345	268 (78)	29 (83)	57 (77)	106 (73)	76 (84)	0.251	n/a
Recurrent depression (MINI)	*n* (%) yes	344	135 (39)	10 (29)	31 (41)	57 (40)	37 (41)	0.583	n/a
Current depression (MINI)	*n* (%) yes	348	194 (56)	22 (63)	40 (53)	78 (53)	54 (59)	0.615	n/a
Past psychosis (MINI)	*n* (%) yes	346	41 (12)	8 (23)	18 (25)	9 (6)	6 (7)	<0.001	BD-I **>** BSp and BD-II **>** BSp + uMDD
Age of mental illness onset	Mean (s.d.)	281	19.6 (11.5)	16 (9.0)	16.5 (8.6)	20.5 (12.5)	21.7 (11.8)	0.019	BD-II **<** uMDD
Psychiatric admissions	*n* (%) yes	277	22 (7.9)	5 (23.8)	5 (8.2)	9 (7.4)	3 (4.1)	0.055	n/a
N stressful life events (LTE)	Mean (s.d.)	326	5.1 (2.5)	5.4 (2.2)	5.3 (2.9)	5.1 (2.6)	5.0 (2.3)	0.784	n/a
Recent stressful event (LTE)	*n* (%) yes	322	176 (55)	17 (52)	46 (66)	78 (57)	35 (43)	0.035	BD-II **>** uMDD
Childhood trauma (CTQ)	Mean (s.d.)	321	42.5 (16.6)	52.1 (16.4)	43.2 (16.1)	40.9 (15.8)	40.8 (17.3)	0.004	BD-I **>** BSp + uMDD
Current GAD (MINI)	*n* (%) yes	348	240 (69)	26 (74)	55 (73)	107 (73)	52 (57)	0.044	none
Current other anxiety (MINI)	*n* (%) yes	330	193 (58)	23 (66)	48 (66)	72 (54)	50 (57)	0.297	n/a
Current OCD (MINI)	*n* (%) yes	347	69 (20)	11 (31)	23 (31)	26 (17)	9 (10)	0.002	BD-I + BD-II **>** uMDD
Current PTSD (MINI)	*n* (%) yes	346	47 (14)	10 (29)	10 (14)	13 (9)	14 (15)	0.020	BD-I **>** BSp
BPD traits (SCID-II)	*n* (%) yes	345	54 (16)	16 (46)	19 (26)	14 (10)	5 (6)	<0.001	BD-I + BD-II **>** BSp + uMDD
PD traits (SAPAS)	Mean (s.d.)	326	3.5 (1.7)	4.1 (1.7)	4.2 (1.7)	3.3 (1.6)	3.1 (1.7)	<0.001	BD-I **>** uMDD and BD-II **>** BSp + uMDD
Substance/alcohol abuse (MINI)	*n* (%) yes	348	88 (25)	11 (31)	27 (36)	36 (24)	14 (15)	0.018	BD-II **>** uMDD
Alcohol use (AUDIT)	Mean (s.d.)	324	7.4 (6.8)	9.8 (9.0)	8.1 (7.4)	7.7 (6.6)	5.4 (4.9)	0.007	BD-I **>** uMDD
Anorexia/bulimia (MINI)	*n* (%) yes	343	17 (5)	1 (3)	8 (11)	5 (3)	3 (3)	0.113	n/a
Suicidality (MINI)	*n* (%) no *n* (%) low *n* (%) medium *n* (%) high	348	144 (41) 144 (41) 44 (13) 16 (5)	7 (20) 19 (54) 6 (17) 6 (9)	29 (39) 30 (40) 10 (13) 6 (8)	71 (48) 53 (36) 18 (12) 5 (3)	37 (41) 42 (46) 10 (11) 2 (2)	0.022	BD-I **>** BSp
Self-criticism reassurance	Mean (s.d.)	325	15.0 (6.1)	15.2 (6.0)	14.9 (6.4)	14.5 (5.8)	15.9 (6.2)	0.355	n/a
Self-criticism negative cognitions	Mean (s.d.)	325	28.7 (12.2)	31.1 (11.1)	31.2 (12.5)	28.8 (11.8)	25.5 (12.5)	0.018	BD-II **>** uMDD
Illness perception score (IPQ)	Mean (s.d.)	318	47.7 (10.1)	50.2 (9.2)	50.0 (7.8)	46.7 (9.95)	46.2 (11.9)	0.034	none
Antidepressant medications	*n* (%) yes	348	158 (45)	16 (46)	36 (48)	66 (45)	40 (44)	0.961	n/a
Mood stabiliser or antipsychotic medications	*n* (%) yes	348	13 (3.7)	1 (2.9)	2 (2.7)	6 (4.1)	4 (4.4)	0.952	n/a

BD-I, bipolar disorder type 1; BD-II, bipolar disorder type 2; BSp, bipolar spectrum; uMDD, unipolar major depressive disorder; MINI, Mini-International Neuropsychiatric Interview; LTE, List of Threatening Events; CTQ, Childhood Trauma Questionnaire; BPD, borderline personality disorder; SCID-II, Structured Clinical Interview for DSM-IV Personality Disorders; PD, personality disorder; SAPAS, Standardised Assessment of Personality – Abbreviated Scale; AUDIT, alcohol use disorders identification test; IPQ, illness perception questionnaire; s.d., standard deviation.

Note that because a single individual can meet the criteria for several diagnoses, the sum of frequencies of comorbidities can be greater than the number of patients.

Participants with BD-I/BD-II had a higher incidence of lifetime psychosis (Fisher's exact test, *p* < 0.001), for both BD-I and BD-II compared to BSp (*p* = 0.013 and *p* < 0.001 respectively), and BD-II compared to uMDD (*p* = 0.007). BD-I patients reported higherchildhood trauma scores compared to BSp (*F*_3,317_ = 4.598, *p* = 0.004; BD-I *v*. BSp, *p* = 0.003) and uMDD (*p* = 0.005) groups. BD-II patients had a younger age of psychiatric symptom onset than those with uMDD (*F*_3,277_ = 3.379, *p* = 0.019; BD-II *v.* uMDD, *p* = 0.039), and were more likely to have had a negative life event in the past one year (χ^2^(3, 322) = 8.615, *p* = 0.035; BD-II *v.* uMDD, *p* = 0.027).

**Fig. 2.**
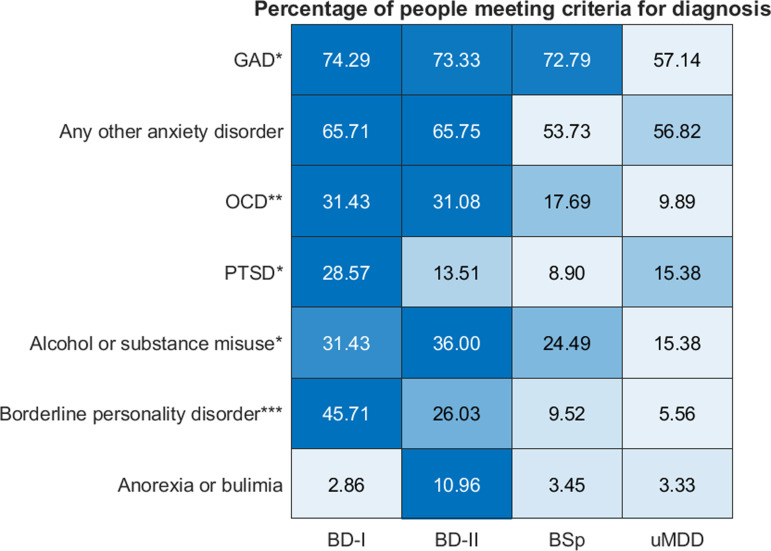
Heatmap depicting the percentage of people meeting criteria for several psychiatric co-morbidities. * *p* < 0.05. ** *p* < 0.01. *** *p* < 0.001. Rows depict each comorbidity; columns depict group. Colours are scaled by row. There were significant differences in the proportions of people meeting the criteria for generalised anxiety disorder (GAD; χ^2^(3, 348) = 8.078, *p* = 0.044; multiple comparisons testing was not significant), obsessive compulsive disorder (OCD; χ^2^(3, 347) = 14.903, *p* = 0.002; BD-I *v.* uMDD, *p* = 0.018; BD-II *v.* uMDD, *p* = 0.004), post-traumatic stress disorder (PTSD; Fisher's exact test, *p* = 0.027; BD-I *v.* BSp, *p* = 0.010), alcohol or substance misuse (χ^2^(3, 348) = 10.027, *p* = 0.018; BD-II *v.* uMDD, *p* = 0.013), and borderline personality disorder (χ^2^(3, 345) = 41.402, *p* < 0.001; BD-I *v.* BSp, *p* < 0.001 and BD-I *v.* uMDD, *p* < 0.001; BD-II *v.* BSp, *p* = 0.007 and BD-II *v.* uMDD, *p* = 0.001). There were no significant differences in the proportion of people meeting the criteria for any other anxiety disorder (including social phobia, agoraphobia or panic disorder; χ^2^(3, 330) = 3.690, *p* = 0.297), nor anorexia or bulimia (Fisher's exact test, *p* = 0.113).

Participants with BD-I/BD-II were more likely to meet the criteria for several other psychiatric diagnoses (see Fig. 2 and Table 2). This was the case for GAD (χ^2^(3, 348) = 8.078, *p* = 0.044), OCD (χ^2^(3, 347) = 14.903, *p* = 0.002) and PTSD (Fisher's exact test, *p* = 0.029). Clinically significant traits of BPD (χ^2^(3, 345) = 41.042, *p* < 0.001) were more frequently present in both BD-I and BD-II groups compared to BSp (*p* < 0.001 and *p* = 0.007 respectively) and uMDD (*p* < 0.001 and *p* = 0.001) groups. This pattern was also seen in trait scores for personality disorders more broadly (*F*_3,322_ = 7.935, *p* < 0.001), where BD-II participants scored higher than both BSp (*p* = 0.002)and uMDD (*p* < 0.001) participants, and BD-I than uMDD (*p* = 0.014). MINI substance or alcohol abuse criteria were met by more BD-II than uMDD participants (χ^2^(3, 348) = 10.027, *p* = 0.018; BD-II *v.* uMDD, *p* = 0.013). BD-I participants reported more excessive alcohol use as measured by the AUDIT questionnaire compared to uMDD participants (*F*_3,320_ = 4.137, *p* = 0.007; BD-I *v.* uMDD, *p* = 0.009).

Participants identified as having bipolar symptomatology scored higher than those with uMDD on several auxiliary psychiatric severity markers. BD-I patients scored higher than BSp patients for suicidality severity (Kruskal-Wallis test, H(3) = 9.610, *p* = 0.022; BD-I *v.* BSp, *p* = 0.019) and BD-II patients scored higher than uMDD patients on measurements of negative cognitions (self-criticism and self-hate; *F*_3,321_ = 3.405, *p* = 0.018; BD-II *v.* uMDD, *p* = 0.020).

#### Multivariate analyses

To explore possible predictors for bipolarity in this population, multinomial logistic regression compared participants with either BD-I or BD-II (BD) *v.* BSp *v.* uMDD. The two BD groups were merged given the comparatively small group size of BD-I and the absence of differences identified in univariate analyses between BD-I and BD-II.

Measures that were statistically significant in the univariate analyses, without small cell sizes, were examined for multicollinearity using a correlation matrix (Online Supplement 3). Those that were highly colinear, with Pearson's *r* > 0.400 (SAPAS, AUDIT and self-criticism), were not included in the logistic regression. Age of symptom onset and childhood trauma scores were negatively correlated with one another (*r* = −0.244, *p* < 0.001) and considered conceptually related; therefore, only age of onset was included in the regression as this would be simpler to assess in routine clinical practice. Variables included in the final logistic regression model were therefore age of onset of psychiatric symptoms; the presence of the substance and/or alcohol abuse, PTSD, and recent negative life events.

The logistic regression outperformed an intercept-only model (χ^2^(8, 258) = 391.10, *p* < 0.001) and was well-fitted to the data (*p* = 0.076 based on deviance criteria). Overall, the model correctly predicted 47.3% of diagnoses, with most errors occurring due to misclassification of BD to BSp (34.6% of errors) and uMDD to BSp (39.7%). Specifically, a younger age of onset of psychiatric symptoms (*p* = 0.009), substance/alcohol abuse (*p* = 0.002) and recent negative life events (*p* = 0.031) distinguished BD from uMDD patients, but PTSD did not (*p* = 0.777). Only recent negative life events (*p* = 0.036) discriminated BSp from uMDD patients. A younger age of onset (*p* = 0.022) and the presence of PTSD (*p* = 0.021) differentiated between BD and BSp participants, while alcohol/substance abuse (*p* = 0.070) and recent negative life events (*p* = 0.754) were not significant. The odds ratios for the individual predictors are shown in Online Supplement 4.

### Therapy outcomes

No between-group differences were identified, either in the rate of therapy receipt (ranging from 83% [BD-I] to 91% [uMDD]; *p* = 0.465), the type of therapy received (*p* = 0.992) or the number of sessions attended (mean 8.98 [s.d. 6.63]; *p* = 0.808, see Online Supplement 5a).

PHQ-9, GAD-7 and WSAS scores before therapy and after therapy, together with the change in therapy scores from before to after therapy, were compared between groups using MANOVA for PHQ-9, GAD-7 and WSAS scores combined. There were no significant differences in pre-therapy scores (one-way MANOVA, *F*_9,704_ = 1.312, *p* = 0.227; Wilk's Λ = 0.960, partial *η*^2^ = 0.013), post-therapy scores (one-way MANOVA, *F*_9,694_ = 1.651, *p* = 0.097; Wilk's Λ = 0.949, partial *η*^2^ = 0.017) or change in scores from pre- to post-therapy (Online Supplement 5b; one-way MANOVA, *F*_9,679_ = 1.056, *p* = 0.393; Wilk's Λ = 0.967, partial *η*^2^ = 0.011). All groups showed a significant decrease in PHQ-9, GAD-7 and WSAS scores (one-way MANOVA effect of intercept, *F*_3,279_ = 41.838, *p* < 0.001; Wilk's Λ = 0.690, partial *η*^2^ = 0.310; effect of intercept on change in PHQ-9, GAD-7 and WSAS scores, *p* < 0.001).

## Discussion

NHS England's IAPT programme aims to provide therapies to individuals affected by uMDD and anxiety disorders and is not widely available to people with SMI – including BD – who are thought to require intervention in more specialist services. Our data suggest a high rate of bipolar spectrum disorders in this naturalistic sample of adults presenting to IAPT and corroborate emerging reports suggesting that individuals accepted for IAPT therapy have complex presentations and meet diagnostic criteria for a range of mental health conditions (Hepgul et al., 2016). The equivalent recovery rates following therapy, regardless of putative diagnosis, could suggest that IAPT may provide appropriate non-pharmacological intervention to some individuals with a bipolar diathesis, at least in the short-term.

### BD prevalence within other vulnerable populations

Overall, the existing evidence suggests that around 50% of people with a provisional uMDD diagnosis may be on the bipolar spectrum: several studies have reported rates of unrecognised (DSM defined) BD-I and/or BD-II of 14–19% (Angst et al., 2011; Hantouche et al., 1998; Smith et al., 2011) and broader bipolarity (including various subthreshold BD definitions) of 39–54% (Angst et al., 2011, 2010; Smith et al., 2011; Zimmermann et al., 2009). However, particularly low rates have been reported when employing more stringent diagnostic assessments e.g., 7.3% of people prescribed antidepressants (Hughes et al., 2016) or 9.6% with uMDD (Smith et al., 2011). Other studies have reported markedly higher rates in outpatients with uMDD, such as 45% meeting DSM criteria for BD-I/II (Benazzi, 1997) or 61% when incorporating subthreshold bipolarity (Benazzi & Akiskal, 2003).

Studies estimating BD prevalence in broader vulnerable populations report similar rates. Manning, Haykal, Connor, and Akiskal (1997) reported that 26% of consecutive primary care patients with anxiety or depression met the criteria for either BD-I, BD-II or cyclothymic disorder. Hosang, Cardno, Freeman, and Ronald (2017) found 47% of a non-clinical adolescent sample exceeded screening thresholds for bipolarity, falling to 9% when employing criteria closer to DSM-defined BD.

Our findings suggest higher rates of bipolarity in an ostensibly ‘non-bipolar’ sample than previous studies. Reasons for this could include the absence of a similar psychological therapies service for people with BD; that individuals can self-refer to IAPT; and it is known that people more often seek help for symptoms of anxiety and depression than hypomania (Hirschfeld et al., 2003). Additionally, these rates may be overestimated and/or could potentially include a minority with a suspected or diagnosed BD (expanded on below).

### Features of under-recognised bipolarity

We found that patients meeting the criteria for BD-I/II presented with more extensive past psychiatric histories and frequently met the criteria for other psychiatric disorders. Concordant with our findings, BD participants in the landmark BRIDGE study had an earlier average age of psychiatric symptom onset, and were more likely to meet the criteria for personality and substance use disorders. We additionally identified characteristics of bipolarity which were not assessed in BRIDGE (higher self-criticism, childhood trauma, rates of recent stressful events and post-traumatic stress disorder) and those which were not significant in BRIDGE (higher suicidality and a history of psychosis). This is however in line with other evidence of increased self-criticism (Forty et al., 2008), childhood trauma histories (McCraw & Parker, 2017), recent stressful life events and PTSD comorbidity (McCraw & Parker, 2017). It is worth noting that the wider literature also supports our findings of increased rates of psychosis (Forty et al., 2008), hospitalisation (Endicott et al., 1985), suicidality (Endicott et al., 1985), comorbid anxiety (Zimmermann et al., 2009), personality disorders (Endicott et al., 1985), substance use disorders (Smith et al., 2011; Zimmermann et al., 2009) and a youngerage of psychiatric symptom onset (Hantouche et al., 1998; Smith et al., 2011). We note that heterogeneity between studies' findings are perhaps exacerbated by heterogeneous study populations.

Our findings of higher tendencies for self-criticism and a higher prevalence of recent negative events support theories that people with BD often have high levels of dysfunctional assumptions resulting in over-ambitious goal setting, perfectionist expectations of reaching goals, and high self-criticism when goals are not achieved or when negative events occur (Alloy et al., 2009). Correspondingly, studies have found that levels of self-criticism interact with negative life events to produce more severe depressive symptoms in participants with bipolar symptoms (Francis-Raniere, Alloy, & Abramson, 2006). This pattern does appear to apply to people with subthreshold bipolarity, and in those at risk for BD can predict diagnostic conversion (Alloy et al., 2009). We were not able to test the specific interactions in this study between self-criticism, negative events and affective symptoms, nor their contributory effects on psychological therapy outcomes. However, participants in our sample with higher self-criticism scores were more likely to have had a recent stressful life event and had more severe affective symptoms, although recent events were not directly associated with symptoms.

A discrepancy between the previous and current studies relates to recurrent depression, which was more prevalent in BD than uMDD patients across several cohorts (Angst et al., 2011; Benazzi & Akiskal, 2003; Forty et al., 2008; Smith et al., 2011), while we found no significant difference between groups. Interestingly, rates of recurrent depression were numerically lower for our BD-I categorised participants in this assessment (29% as opposed to ~40% in other participant groups). The rates of recurrent depression in the current sample were also relatively low compared to previous studies (Angst et al., 2011; Benazzi & Akiskal, 2003), likely because only 78% of our participants overall met the criteria for lifetime depression and that overall, around 65% of those with depression will have recurrent episodes (Eaton et al., 2008).

### The ‘spectrum’ group

The HCL (a participant-rated screening tool) clearly differs from the MINI (an investigator-rated interview aligning with diagnostic criteria) and thus it is unsurprising that some score positively for bipolarity on the former but not the latter. Notably, the MINI requires participants to answer positively to questions pertaining to (hypo)manic symptoms being impairing/unusual and of a certain duration. Although the HCL also includes these questions, a participant does not have to answer positively in order to exceed HCL score thresholds.

Notably, the BRIDGE study found greater differences between BSp and uMDD than DSM-BD *v.* uMDD, on several parameters (e.g. anxiety, personality and substance use disorders). In our study, these factors differed most between those with DSM-BD and either BSp or uMDD. Strikingly, we did not identify any significant differences between BSp and uMDD participants, which could suggest that the HCL is oversensitive to bipolarity. Despite promise from the original HCL validation (Forty et al., 2010), others have found the HCL to frequently display false positive BD cases (Smith et al., 2011). Literature syntheses indicate that the HCL has a sensitivity of 82% and specificity of 57% for diagnosing substantive BDs (Wang et al., 2019), although there is limited data available in primary care and community settings. We therefore recommend the HCL is applied in non-vulnerable as well as vulnerable cohorts, and in general practice settings. We emphasise that this is a screening, not diagnostic, tool and there are concerning implications of both false-positive and false-negative BD diagnoses. While the MINI has comparable sensitivity (81%) and higher specificity (94%) than the HCL (against the SCID; Sheehan et al., 1997), it is a rapid diagnostic interview and also may be over-inclusive with BD diagnoses.

A combination of measurement limitations, the high proportion of BD/BSp participants (totalling 70%) categorised, and the lack of differences illustrated between BSp and uMDD groups together support an over-estimation of bipolar spectrum disorders in our study.

### Sample representativeness

This study was carried out in a single London-based IAPT service, which limits the generalisability of the results. Within this service, our participants comprise <5% of those receiving therapy in Southwark. A summary comparison of our sample's composition, compared with Southwark IAPT's during this time frame, can be found in Online Supplement 6. These data suggest that our sample was representative in terms of age and gender composition. Our sample had a higher proportion of white participants (77%) than the IAPT service during this time (70%), and even the latter is likely to comprise an over-representation of white individuals compared the local area of residents (~50% according to census data). We had considered it possible that people referred into IAPT who did not consent to participate in this study could have had more severe illness than our participants, however a much higher proportion of our sample was depressed at the time of the study session (71% exceeding the PHQ symptom severity threshold, compared to 15% in Southwark IAPT). Our participants had a slightly higher rate of recovery according to IAPT definitions (41%) than the service overall (37%), although this remains numerically lower than the national average at the time (45–46%). We do not have access to information concerning the reasons for our participants not completing therapy. However, participants in this study were more likely to initiate treatment after IAPT referral (88%) than either Southwark or national averages across these two years (71% and 66% respectively) and had a higher average number of therapy sessions compared to the national average (9.0 *v.* 6.9) (IAPT annual report, 2019–2020; not reported for 2014–2016).

### Methodological considerations

We note that therapeutic outcomes were assessed in the short-term only (at the final therapy session) and longer-term response would have provided a more reliable estimate of IAPT suitability for those with bipolar symptoms. We also did not assess manic symptoms or other features of bipolarity e.g., (hypo)mania history, or what difficulty therapy was being sought for (i.e. IAPT ‘problem descriptors’).

We did not assess other potential comorbidities, such as ADHD and personality disorders other than BPD. Exceeding HCL thresholds may, instead of a bipolar spectrum diathesis, indicate one of these – or other assessed – illnesses (Baek, Kim, Nierenberg, Jeon, & Hong, 2020). Regarding comorbidity more broadly, participants indicated to have multiple comorbidities on the measures we administered may, in a comprehensive assessment by a qualified clinician, indicate a single (or fewer) diagnosis. This is particularly poignant for the BSp group, who were categorised according to a non-diagnostic measure. As noted above, we emphasise that the sensitivity and specificity of the bipolar assessments is suboptimal. HCL scores may be raised due to recall bias (e.g. individuals experiencing a depressive episode may ‘over-value’ periods when they were well), while the MINI was administered in this study by non-clinical doctoral researchers (albeit with training and supervision by an experienced psychiatrist). MINI training included structured sessions with an expert in the field (AJC; Chief Investigator) which included role play and video vignettes as well as detailed guidance provided verbally and in writing. The initial training was supplemented by ongoing (monthly) supervision and checking of ratings where uncertainties raised by the study researchers, as well as video recording checked by study academics; however, no formal reliability assessment was undertaken, which is a limitation of the current work. Having a matched, non-IAPT control group would have provided more information about these factors.

Critically, we were unable to ascertain the rate of *diagnosed* BD-I or BD-II in this sample. It is possible that individuals within the sample may have previously received a diagnosis of bipolar disorder, although as this theoretically would have precluded them from being referred to IAPT and based on previous data, this rate is likely to be 0–5% (Richardson, Wrightman, Yeebo, & Lisicka, 2017).

Finally, several measures were examined and in combination with a small sample size in some subgroups of this sample, there is the possibility of type I error. Nevertheless, our work is strengthened by its reasonable overall sample size, adjustment for multiple comparisons, the naturalistic setting, its combination of replicating previous studies and adding novel findings as well as having a putative clinical application.

### Clinical implications

In this sample, depression and anxiety severity after IAPT therapy were reduced on average from moderate to mild severity, similar (or slightly better) in terms of recovery than this service provides as per annual report data. The lack of difference between BD, BSp and uMDD groups in therapy response suggests that these widely available therapies could be appropriate for people with BD. More broadly, the effectiveness of psychological therapies for BD is reflected by prioritisation in clinical guidelines (NICE, 2020) despite concerns about the risk of bias in the evidence (Tong, Strawbridge, & Jauhar, 2021). IAPT pilot programmes have recently been trialled for people with SMI, but despite efforts made to address the low rates of therapy access for severely ill people (Jones et al., 2018), it is not yet known how widely this will be available. Whilst our data encouragingly suggests that patients with bipolar symptoms respond equally well to primary care-based therapy provision, further work is needed to ascertain the validity of this finding (including replication in a representative sample of people with BD diagnoses). However, IAPT may provide appropriate non-pharmacological intervention to some individuals with a bipolar diathesis, at least in the short-term. We consider it important for future research to replicate our methods in other IAPT services. Should these findings be replicated in independent IAPT samples with long-term follow-up of therapy outcomes, implications could include, firstly, an expansion of primary care therapy services accessible to those with BD. Despite the resources required to implement this, it could reduce long-term future costs (Radhakrishnan et al., 2013). There has, thus far, been limited investigation into the suitability of such a (primary care) service for people with BD diagnoses, and we are aware of no considerations about undiagnosed individuals on the bipolar spectrum.

Secondly, IAPT assessments could be resourced to assess risk for bipolarity (with vigilance around patients with anxiety as well as depression), which could be fed back to medical practitioners. This is a controversial issue. Optimistically, it could improve the accuracy of BD diagnoses, the timeliness and appropriateness of multidisciplinary treatment. However, this also could result in one or more of the following: BD indication used to justify referral refusals; a high rate of false-positive findings (if screening tools are used); and/or placing a substantial burden on (already strained) resources to undertake full diagnostic assessments. Any service changes must not be used to reduce access to care and as such, this issue clearly requires further examination.

Additionally, advancing towards a universal consensus on the definition of clinically-relevant BSp would help to facilitate more appropriate treatment and research in this understudied group. Our data shows that such presentations are common, despite usually being under-recognised.

Our findings support the converging evidence illustrating a high rate of undiagnosed BD which can have substantial consequences, such as treatment with monoaminergic medications that are ineffective or can destabilise affect (Hirschfeld, Cass, Holt, & Carlson, 2005). As such, early detection of bipolarity is critical. Screening for bipolar symptoms is supported by the increasingly established markers indicating BD risk. As well as providing a source for potentially valuable BD screening, this study preliminarily suggests promise for already available psychological therapies, for people with a bipolar diathesis. We sincerely hope to stimulate future efforts in refining primary care psychological therapy services so they can support people at risk of severe mental illnesses.

## Supplementary material

For supplementary material accompanying this paper visit https://doi.org/10.1017/S0033291722002343. click here to view supplementary material
