# Supplementary material for: Is there a ‘bipolar iceberg’ in UK primary care psychological therapy services?
Source: Psychol Med. 2022 Aug 3;53(12):5385–94. doi: 10.1017/S0033291722002343 (PMC10482719; doi:10.1017/S0033291722002343)
Supplement: Supplementary file 1 [file S0033291722002343sup001.docx]

**Supplement 1: detail and references of measures administered**

*Sociodemographic characteristics*

Continuous variables comprised age, BMI, physical illness severity (total Modified Cumulative Illness Rating Scale score excluding the mental health item^1^) and the extent of social support available to them (total score from the patient-rated Oslo 3 social support scale^2^). Categorical variables comprised gender (binary; all participants identified as male or female via self-report), ethnicity (nominal), education level (highest qualification; ordinal), relationship status (nominal) and employment status (binary).

### Clinical characteristics

These were pre-selected for inclusion based on existing evidence of associations with bipolarity in addition to data availability from the PROMPT study. Historical factors included:

- Past major depressive episode (MDE, according to the MINI interview^3^)
- Past psychosis (according to the MINI^3^)
- Age of mental illness onset (patient-report)
- Number of psychiatric hospital admissions (lifetime)
- Negative life events (total score from the lifetime List of Threatening Events questionnaire, and binary variable indicating experience of such an event in the last 12 months^4^)
- Childhood trauma severity (total score from the Childhood Trauma Questionnaire^5^)

Current presentation factors included:

- Recurrent MDE ([according to the MINI^3^)
- OCD (according to the MINI^3^)
- PTSD (according to the MINI^3^)
- GAD (according to the MINI^3^)
- Any other anxiety disorder (including panic disorder, agoraphobia and social phobia, according to the MINI^3^)
- Significant traits of borderline personality disorder (BPD; i.e. scoring ‘definite’ for at least five trait items on the BPD subsection of the Structured Clinical Interview for DSM-IV Personality Disorders^6^)
- Traits of personality disorder more broadly (total score from the Standardised Assessment of Personality – Abbreviated Scale [SAPAS]^7^)
- Substance abuse (scoring positively for substance abuse and/or alcohol abuse on the MINI interview^3^)
- Extent of alcohol use (total score on the AUDIT assessment^8^)
- Eating disorder (scoring positively for anorexia and/or bulimia on the MINI interview^3^)
- Suicidality severity (according to MINI interview^3^)
- Self-criticism (negative cognitions and reassurance subscales from the Forms of Self-critical/Attacking and Self-reassuring Scale^9^)
- Illness perception (total score from the illness perception questionnaire^10^)
- Antidepressant use (current)
- Mood stabiliser or antipsychotic medication use (current)

Participants’ HCL scores (measuring self-reported bipolarity)^11^ and the proportion of participants meeting the criteria for a current major depressive episode (MINI interview)^3^ were not examined statistically (because these were associated with the criteria used to define participant group categories) but are reported descriptively.

### Therapy characteristics

Participant-rated wellbeing assessments from the PROMPT research assessment (pre-therapy) and final therapy session attended (post-therapy) were recorded, measuring symptoms of depression (Patient Health Questionnaire [PHQ-9] total score^12^), anxiety (Generalised Anxiety Disorder [GAD-7] total score^13^) and psychosocial functioning (Work and Social Adjustment Scale [WSAS] total score^14^). Also assessed was the proportion of participants receiving therapy (defined as attending two or more sessions), and for therapy completers the number of sessions and type of therapy received.

**References for measures**

1. Salvi F, Miller MD, Grilli A, Giorgi R, Towers AL, Morichi V, et al. A manual of guidelines to score the modified cumulative illness rating scale and its validation in acute hospitalized elderly patients. J Am Geriatr Soc 2008;56(10):1926–31.

2. Dalgard O. Community mental health profile as tool for psychiatric prevention. In: Promotion of mental health. Eds: Trent D. R. and Reed C. Aldershot: Avebury; 1996.

3. Sheehan DV, Lecrubier Y, Sheehan KH, Amorim P, Janavs J, Weiller E, et al. The Mini-International Neuropsychiatric Interview (M.I.N.I.): the development and validation of a structured diagnostic psychiatric interview for DSM-IV and ICD-10. J Clin Psychiatry 1998;59 Suppl 20:22-33.

4. Brugha T, Bebbington P, Tennant C, Hurry J. The List of Threatening Experiences: a subset of 12 life event categories with considerable long-term contextual threat. Psychol Med 1985;15(1):189–94.

5. Bernstein DP, Fink L, Handelsman L, Foote J, Lovejoy M, Wenzel K, et al. Initial reliability and validity of a new retrospective measure of child abuse and neglect. Am J Psychiatry 1994;151(8):1132–6.

6. First MB, Gibbon M, Spitzer RL, Williams JBW, Benjamin LS. Structured Clinical Interview for DSM-IV Axis II Personality Disorders (SCID-II). Washington, DC American Psychiatric Press, 1997.

7. Moran P, Leese M, Lee T, Walters P, Thornicroft G, Mann A. Standardised Assessment of Personality – Abbreviated Scale (SAPAS): Preliminary validation of a brief screen for personality disorder. Br J Psychiatry 2003;183(3):228–32.

8. Babor TF, de la Fuente JR, Saunders J, Grant M. AUDIT: The alcohol use disorders identification test: Guidelines for use in primary health care 1992. http://bases.bireme.br/cgi-bin/wxislind.exe/iah/online/?IsisScript=iah/iah.xis&src=google&base=PAHO&lang=p&nextAction=lnk&exprSearch=14305&indexSearch=ID Accessed September 7, 2021.

9. Castilho P, Pinto-Gouveia J, Duarte J. Exploring self-criticism: confirmatory factor analysis of the FSCRS in clinical and nonclinical samples. Clin Psychol Psychother 2015;22(2):153–64.

10. Weinman J, Petrie KJ, Moss-morris R, Horne R. The illness perception questionnaire: A new method for assessing the cognitive representation of illness. Psychol Health 1996;11(3):431–45.

11. Forty L, Kelly M, Jones L, Jones I, Barnes E, Caesar S, et al. Reducing the Hypomania Checklist (HCL-32) to a 16-item version. J Affect Disord 2010;124(3):351–6.

12. Kroenke K, Spitzer RL. The PHQ-9: A New Depression Diagnostic and Severity Measure. Psychiatr Ann 2002;32(9):509–15.

13. Spitzer RL, Kroenke K, Williams JBW, Löwe B. A brief measure for assessing generalized anxiety disorder: the GAD-7. Arch Intern Med 2006;166(10):1092–7.

14. Mundt JC, Marks IM, Shear MK, Greist JH. The Work and Social Adjustment Scale: a simple measure of impairment in functioning. Br J Psychiatry 2002;180:461–4.

### **Supplement 2. Full statistical description of univariate comparisons of clinical-related variables between four participant groups**

**A** ANOVA analyses with Tukey’s HSD

| **Characteristic** | **F statistic** | **p value** | **BD-1 vs BSp** | **BD-1 vs uMDD** | **BD-2 vs BSp** | **BD-2 vs uMDD** |
| --- | --- | --- | --- | --- | --- | --- |
| **Age of mental illness onset** | *F*_3,277_ = 3.379 | 0.019 | ~ | ~ | ~ | p = 0.039 |
| **N stressful life events (LTE)** | *F*_3,322_ = 0.356 | 0.784 |  |  |  |  |
| **Childhood trauma (CTQ)** | *F*_3,317_ = 4.598 | 0.004 | p = 0.003 | p = 0.005 | ~ | ~ |
| **PD traits (SAPAS)** | *F*_3,322_ = 7.935 | <0.001 | ~ | p = 0.014 | p = 0.002 | p < 0.001 |
| **Alcohol use (AUDIT)** | *F*_3,320_ = 4.137 | 0.007 | ~ | p = 0.009 | ~ | ~ |
| **Self-criticism reassurance** | *F*_3,321_ = 1.086 | 0.355 |  |  |  |  |
| **Self-criticism negative cognitions** | *F*_3,321_ = 3.405 | 0.018 | ~ | ~ | ~ | p = 0.020 |
| **Illness perception score (IPQ)** | *F*_3,314_ = 2.925 | 0.034 | ~ | ~ | ~ | ~ |

Grey cells. Overall (4-way) test statistics. In all cases except age of mental illness onset (blue), the variable is higher in the first group. ~ indicates the comparison was not significant.

* NB no differences significant between BD-1 and BD-2 participants or BSp and uMDD participants (no column for their comparisons)

**B** Chi-square, Fisher’s exact test and Kruskall-Wallis tests with Bonferroni correction

| **Characteristic** | **Relevant statistic** | **p value** | **BD-1 vs BSp** | **BD-1 vs uMDD** | **BD-2 vs BSp** | **BD-2 vs uMDD** |  |
| --- | --- | --- | --- | --- | --- | --- | --- |
| **Past depression (MINI)** | *X^2^*_(3)_ = 4.099 | 0.251 |  | | | |  |
| **Recurrent depression (MINI)** | *X^2^*_(3)_ = 1.948 | 0.583 |  | | | |  |
| **Past psychosis (MINI)** | *X^2^*_(3)_ = 22.547 | <0.001 | p = 0.013 | ~ | p < 0.001 | p = 0.007 |  |
| **Past psychiatric admission** | Fisher’s exact test | 0.053 |  | | | |  |
| **Recent stressful event (LTE)** | *X^2^*_(3)_ = 8.615 | 0.035 | ~ | ~ | ~ | p = 0.027 |  |
| **Current GAD (MINI)** | *X­^2^*_(3)_ = 8.078 | 0.044 | ~ | ~ | ~ | ~ |  |
| **Current other anxiety (MINI)** | *X^2^*_(3)_ = 3.690 | 0.297 |  | | | |  |
| **Current OCD (MINI)** | *X*^2^_(3)_ = 14.903 | 0.002 | ~ | p = 0.018 | ~ | p = 0.004 |  |
| **Current PTSD (MINI)** | Fisher’s exact test | 0.027 | p = 0.010 | ~ | ~ | ~ |  |
| **BPD traits (SCID-II)** | *X*^2^_(3)_ = 41.042 | <0.001 | p < 0.001 | p < 0.001 | p = 0.007 | p = 0.001 |  |
| **Substance/alcohol abuse (MINI)** | *X*^2^_(3)_ = 10.027 | 0.018 | ~ | ~ | ~ | p = 0.013 |  |
| **Anorexia/bulimia (MINI)** | Fisher’s exact test | 0.113 |  | | | |  |
| **Suicidality (MINI)** | H_(3)_ = 9.610 | 0.022 | 0.019 | ~ | ~ | ~ |  |
| **Antidepressant medications** | *X*^2^_(3)_ = 0.297 | 0.961 |  | | | |  |
| **Mood stabiliser/antipsychotic medications** | Fisher’s exact test | 0.952 |  | | | |  |

Grey cells. Overall (4-way) test statistics. In all cases, the proportion is higher in the first group. ~ indicates the comparison was not significant.

* NB no differences significant between BD-1 and BD-2 participants or BSp and uMDD participants (no column for their comparisons)

### **Supplement 3. Correlation matrix of multi-collinearity assessment of variables in the multinomial logistic regression.**

| **Correlation matrix (Pearson's r)** | **Alcohol/ substance abuse** | **Post-traumatic stress disorder** | **Recent negative life events** | **Age of psychiatric symptom onset** |
| --- | --- | --- | --- | --- |
| **Substance/alcohol abuse** | 1.000 | 0.047 | 0.134* | -0.103 |
| **Post-traumatic stress disorder** | 0.047 | 1.000 | 0.020 | -0.025 |
| **Recent negative life events** | 0.134* | 0.020 | 1.000 | -0.083 |
| **Age of psychiatric symptom onset** | -0.103 | -0.025 | -0.083 | 1.000 |

Pearson’s correlation (r) between variables included in the multinomial model. * p < 0.05. ** p < 0.01.

**Supplement 4. Between-group comparisons for variables used in the multinomial logistic regression.**

|  | **BD vs BSp** | **BD vs uMDD** | **BSp vs uMDD** |
| --- | --- | --- | --- |
| **Substance/alcohol abuse** | OR: 1.813 (0.952 to 3.453)  p = 0.070 | OR: 4.183 (1.716 to 10.195)  p = 0.002 | OR: 2.307 (0.968 to 5.498)  p = 0.059 |
| **Post-traumatic stress disorder** | OR: 3.177 (1.192 to 8.469)  p = 0.021 | OR: 1.152 (0.433 to 3.065)  p = 0.777 | OR: 0.363 (0.125 to 1.050)  p = 0.062 |
| **Recent negative life events** | OR: 1.102 (0.599 to 2.030)  p = 0.754 | OR: 2.167 (1.072 to 4.379)  p = 0.031 | OR: 1.966 (1.046 to 3.694)  p = 0.036 |
| **Age of psychiatric symptoms onset** | OR: 0.966 (0.937 to 0.995)  p = 0.022 | OR: 0.957 (0.927 to 0.989)  p = 0.009 | OR: 0.992 (0.967 to 1.017)  p = 0.505 |

Odds ratios (and 95% confidence intervals) for predictor variables. Dark orange = p < 0.05; lighter orange = p < 0.1; grey = non-significant.

**Supplement 5: Therapy characteristics and outcome**

### 5a. Therapy characteristics

| **Characteristic** | |  | **n** | **ALL** | **BD-I** | **BD-II** | **BSp** | **uMDD** |
| --- | --- | --- | --- | --- | --- | --- | --- | --- |
| **Received therapy** | | n (%) yes | 343 | 301 (88) | 29 (83) | 61 (85) | 129 (88) | 82 (91) |
| **Number of therapy sessions** | | Mean (SD) | 343 | 9.0 (6.6) | 8.9 (6.5) | 8.3 (6.2) | 9.2 (7.2) | 9.3 (6.2) |
| **Type of therapy** | **CBT**  **Counselling**  **Group self-help**  **Other** | n (%) yes | 246 | 94 (38)  56 (23)  67 (27)  29 (12) | 8 (36)  5 (23)  6 (27)  3 (14) | 23 (44)  12 (23)  11 (21)  6 (12) | 40 (37)  25 (23)  30 (28)  13 (12) | 23 (36)  14 (22)  20 (31)  7 (11) |
| **Depressive symptoms (PHQ-9):**  **pre-therapy**  **post-therapy** | | Mean (SD)  Mean (SD) | 297  298 | 14.1 (6.5)  9.6 (6.9) | 15.4 (6.9)  9.1 (7.0) | 14.3 (6.4)  10.2 (7.0) | 13.6 (6.7)  9.1 (6.6) | 14.4 (6.3)  10.3 (7.5) |
| **Anxiety symptoms (GAD-7):**  **pre-therapy**  **post-therapy** | | Mean (SD)  Mean (SD) | 297  298 | 11.9 (5.6)  8.1 (6.0) | 14.3 (4.8)  9.2 (5.7) | 12.2 (5.5)  8.7 (6.1) | 11.5 (5.7)  7.4 (5.5) | 11.4 (5.7)  8.3 (6.6) |
| **Psychosocial functioning (WSAS):**  **pre-therapy**  **post-therapy** | | Mean (SD)  Mean (SD) | 295  285 | 18.8 (9.1)  14.4 (9.7) | 20.0 (10.3)  13.5 (9.9) | 19.1 (7.4)  16.9 (9.5) | 18.8 (8.7)  13.3 (9.1) | 18.2 (10.3)  14.4 (10.5) |

Receipt and response to IAPT therapy between groups.

Abbreviations: BD-I = bipolar disorder type 1, BD-II = bipolar disorder type 2, BSp = bipolar spectrum, uMDD = unipolar major depressive disorder, SD = standard deviation, CBT = cognitive behavioural therapy, PHQ-9 = 9-item patient health questionnaire of depressive symptoms^21^, GAD-7 = 7-item generalised anxiety disorder questionnaire^22^, WSAS = work and social adjustment scale^23^.


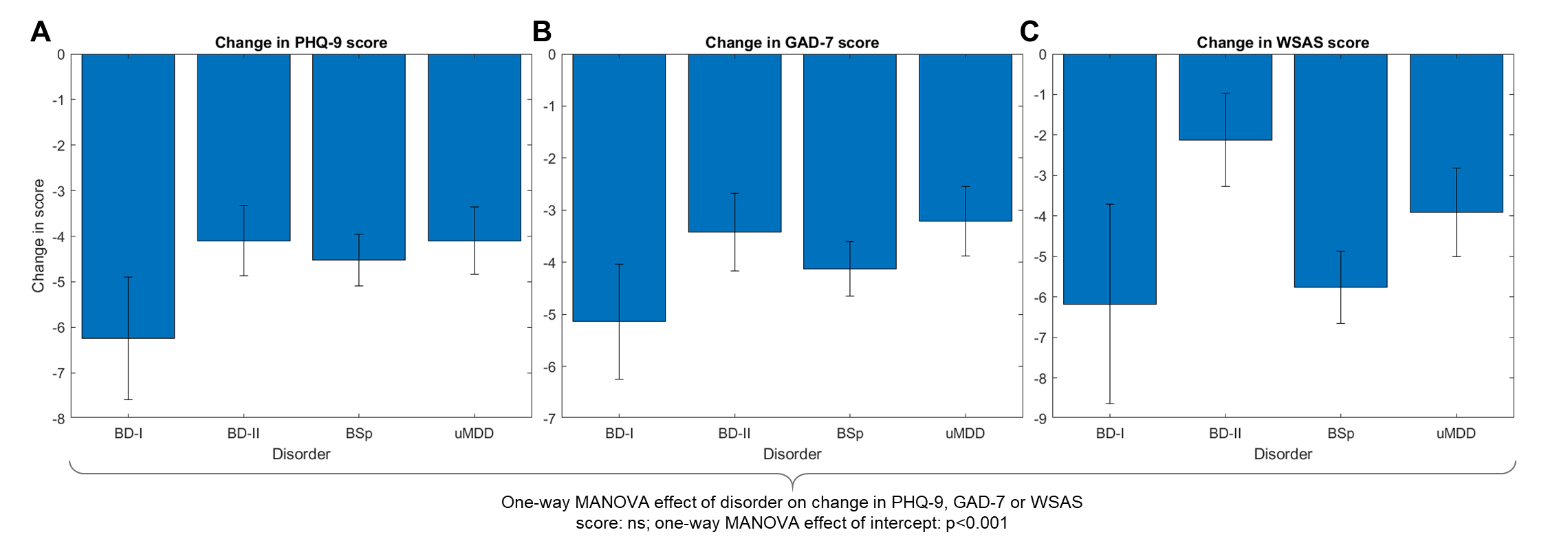


**5b. Change in PHQ-9, GAD-7 and WSAS scores from pre- to post-therapy in people who had received at least two sessions of therapy.**

Error bars show standard error of the mean. Multivariate analysis of variance (MANOVA) showed that there was no effect of disorder (BD-I v BD-II v BSp v uMDD) on the change in either **A** PHQ-9 scores, **B** GAD-7 scores, or **C** WSAS scores (one-way MANOVA, *F*_9,679_ = 1.056, p = 0.393; Wilk's Λ = 0.967, partial η^2^ = 0.011). Across groups, there was a significant decrease in PHQ-9, GAD-7 and WSAS scores compared to zero (one-way MANOVA effect of intercept, *F*_3,279_= 41.838, p < 0.001; Wilk's Λ = 0.690, partial η^2^ = 0.310; effect of intercept for change in PHQ-9, GAD-7 and WSAS scores, p < 0.001)

**Supplement 6: Sample comparison with Southwark IAPT as a whole**

From annual IAPT reports covering the time period April 2014 – March 2016 (i.e., slightly shorter than the PROMPT study), this IAPT service provided therapy to 7,165 individuals [371 PROMPT participants = 5.2%].

| **Group described** | **n patients** | **% depressed pre-therapy** | **% recovered** | **% female** | **Average age** | **% white** |
| --- | --- | --- | --- | --- | --- | --- |
| 2014-2015 Southwark IAPT | 3555 | 15% (PHQ) | 37% of all cases ^a^ | 67% | ~36 ^b^ | 70% |
| 2015-2016 Southwark IAPT | 3610 | 15% (PHQ) | 37% of all cases ^a^  Average PHQ score (depressed cases at baseline) 17.5 pretherapy - 12.3 post-therapy | 66% | ~36 ^b^ | 70% |
| Our sample | 371 | 56% (MINI)  71% PHQ | 41% of all cases ^a^  Average PHQ change (all patients) from 14.1 to 9.6  Average PHQ change (PHQ cases) from 17.0 to 11.22 | 63% | 39.6 | 77% |

^a^ recovery defined as meeting criteria for a clinical case (depression or anxiety) before therapy and not meeting criteria for a case on either depression or anxiety measure after treatment.

^b^ Estimate: Almost identical number of participants aged 18-35 as 36-64 (with few under 18 or over 65)

**Information retrieved from:**

2014-2015 <https://files.digital.nhs.uk/publicationimport/pub19xxx/pub19098/psych-ther-ann-rep-tab-2014-15.xlsx> and <https://files.digital.nhs.uk/publicationimport/pub19xxx/pub19098/psyc-ther-ann-rep-2014-15.pdf>

2015-2016 <https://files.digital.nhs.uk/excel/1/q/psych-ther-ann-rep-tab-2015-16-v2.xlsx> and <https://files.digital.nhs.uk/pdf/1/0/psyc-ther-ann-rep-2015-16_v2.pdf>
